# Comparative Analysis of Volatile Organic Compounds in Different Parts of Ginseng Powder Using Gas Chromatography–Ion Mobility Spectrometry

**DOI:** 10.3390/molecules30091965

**Published:** 2025-04-29

**Authors:** Manshu Zou, Ximing Yu, Yuhuan Liu, Lijun Zhu, Feilin Ou, Chang Lei

**Affiliations:** State Key Laboratory of Chinese Medicine Powder and Medicine Innovation in Hunan (Incubation), Science and Technology Innovation Center, Hunan University of Chinese Medicine, Changsha 410208, China; zoumanshu@hnucm.edu.cn (M.Z.); 004016@hnucm.edu.cn (X.Y.); yuhuan-liu@stu.hnucm.edu.cn (Y.L.); 202104030124@stu.hnucm.edu.cn (L.Z.); fl25800122@126.com (F.O.)

**Keywords:** ginseng, different parts, volatile organic compounds, gas chromatography–ion mobility spectrometry, Euclidean distance, principal component analysis, partial least-squares discriminant analysis, cluster analysis

## Abstract

The main root, reed head, and fibrous root are three different main edible medicinal parts of ginseng (*Panax ginseng* C. A. Meyer). When processed into ginseng products, such as ginseng powder, they exhibit similar colors and odors, easily confused in market circulation. However, there are differences in their pharmacological activity and clinical indications. Therefore, the identification of the different parts of ginseng powder is crucial for ensuring the quality, safety, and efficacy of medicinal ginseng products. In this study, we utilized gas chromatography–ion mobility spectrometry (GC–IMS) to analyze volatile organic components (VOCs) in main root, reed head, and fibrous root of ginseng. It was found that the composition of VOCs in different parts of ginseng powder was similar, but the content was different in all samples, and a total of 68 signal peaks was detected and 65 VOCs identified. In addition, combined with fingerprint analysis, principal component analysis (PCA), Euclidean distance, partial-least squares discriminant analysis (PLS-DA), and cluster analysis (CA), it clearly showed the significant differences between VOCs in different parts of ginseng powder. Our findings reveal that GC–IMS combined with chemometrics is a reliable method for distinguishing the active parts of ginseng powder, and provides essential data support for different parts of ginseng processing and functional product development.

## 1. Introduction

Ginseng (*Panax ginseng* C. A. Meyer) is a functional food primarily found in China, North Korea, and Japan. According to its place of origin, ginseng can be categorized into Jilin ginseng, Liao ginseng, Goryeo ginseng, Japanese ginseng, and so on. Ginseng is extensively utilized as both a common and functional food in China, Japan, North Korea, South Korea, and various other regions globally. Moreover, it is frequently used to enhance immune function [1], prevent cardiovascular and cerebrovascular diseases [2], alleviate fatigue [3], inhibit oxidation [4], delay aging [5], improve memory [6], and promote anti-cancer effects [7]. In 2009, the Codex Alimentarius Commission (CAC) adopted the International Standard for Ginseng Food, following which the Ministry of Health of the People’s Republic of China permitted ginseng cultivation as food in 2012 under Regulation No. 17. The primary edible parts of ginseng are the roots and rhizomes. The rhizome is the head part of the reed (named the reed head), and the root is mainly divided into two parts: the main root and the fibrous root.

It is easy to distinguish the main root, reed head, and fibrous root of ginseng by appearance and form, but ginseng-related foods are usually sold in powder form, and the color and smell of the powder from the three ginseng parts are similar, which is difficult to distinguish with the naked eye or by smell. There is a significant price disparity among the main roots, fibrous roots, and reed head of ginseng, indicating that high-quality ginseng powder primarily consists of the main root powder; the reed head and fibrous roots of ginseng are often made into general-quality ginseng powder due to their lower cost. Some illegal traders exploit this feature to sell fiber root and reed head powder as main root powder for profit, seriously disrupting the market order. Studies have demonstrated that these parts contain many similar active ingredients, such as ginsenosides [8]. The average value of the total content of 10 ginsenosides (Rb_1_, Rb_2_, Rb_3_, Rc, Rd, Re, Rf, Rg_1_, Rg_2_, and Ro) follows the order: ginseng fibrous roots > ginseng reed head > ginseng main roots, indicating that fibrous roots can serve as the primary component when ginseng saponins are utilized as functional constituents. Simultaneously, there is some evidence of the poor quality or adulteration of ginseng products in the market, which may have a negative impact on their quality and safety. At present, high-performance liquid chromatography (HPLC) [8] and gas chromatography-mass spectrometry (GC-MS) [9] can be used as the main method to identify different parts of ginseng powder, but this method is complicated, time-consuming, and destructive to samples. Therefore, it is of practical significance to distinguish and identify different parts of ginseng powder using an efficient method to ensure the safety and efficacy of health foods.

Gas chromatography–olfactometry (GC–O), electronic nose (e-nose), gas chromatography–ion mobility spectrometry (GC–IMS), and other technologies are used to detect volatile organic components (VOCs) and evaluate the authenticity and quality of food. Among them, GC–IMS is a newly developed technique for the analysis and detection of VOCs. GC–IMS combines the high separation capability of gas chromatography with the high sensitivity of ion mobility spectrometry to effectively separate and detect VOCs in samples, even at trace levels [10,11]. GC–IMS has the advantage of a fast response and can qualitatively and quantitatively analyze samples in a short time [12]. The 3D spectra (retention time, drift time, and signal strength) obtained via GC–IMS can visually demonstrate differences in the VOCs in a sample. Direct headspace sampling allows the sample to be analyzed without special treatment or enrichment, saving time and effort while retaining the original flavor of the sample. At present, GC–IMS is a rapid, efficient, convenient, and eco-friendly technique widely used for analyzing VOCs in food and flavor analysis [13,14].

The primary bioactive components of ginseng include ginsenosides [15,16], polysaccharides [17], and VOCs [9,18]. VOCs are significant active substances in plant-based foods. A small dose of VOCs in ginseng exhibits excitatory effects, a moderate dose induces sedative effects, and a high dose results in paralysis [19]. Moreover, VOCs exhibit antibacterial, anti-tumor, and myocardial ischemia-alleviating effects [20,21]. The utilization of GC–IMS for analyzing VOCs in different parts of ginseng (the main root, reed head, and fibrous root) powder holds significant importance in selecting suitable parts to develop products. Although, some researchers have indicated that the types and contents of ginsenosides differ in different parts of ginseng [8], but a study of VOCs in different parts of ginseng powder has not been reported. In this study, we employed the GC–IMS method to rapidly analyze VOCs in different parts of ginseng powder and to establish the fingerprints of VOCs to identify the characteristic substances and further treatment of data by principal component analysis (PCA), cluster analysis (CA), and partial least-squares discriminant analysis (PLS-DA). The results of this study are helpful for providing a reference for the identification, development, and utilization of different parts of ginseng.

## 2. Results

### 2.1. Qualitative Analysis of VOCs in Different Parts of Ginseng Powder

**Figure 1:**
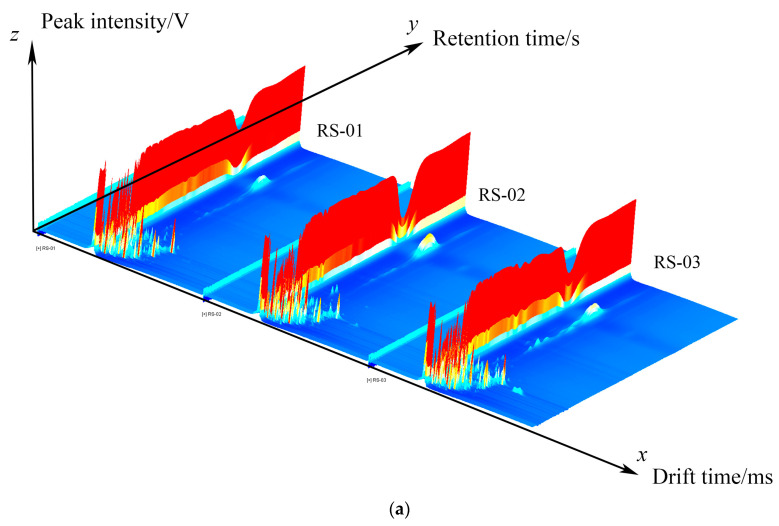

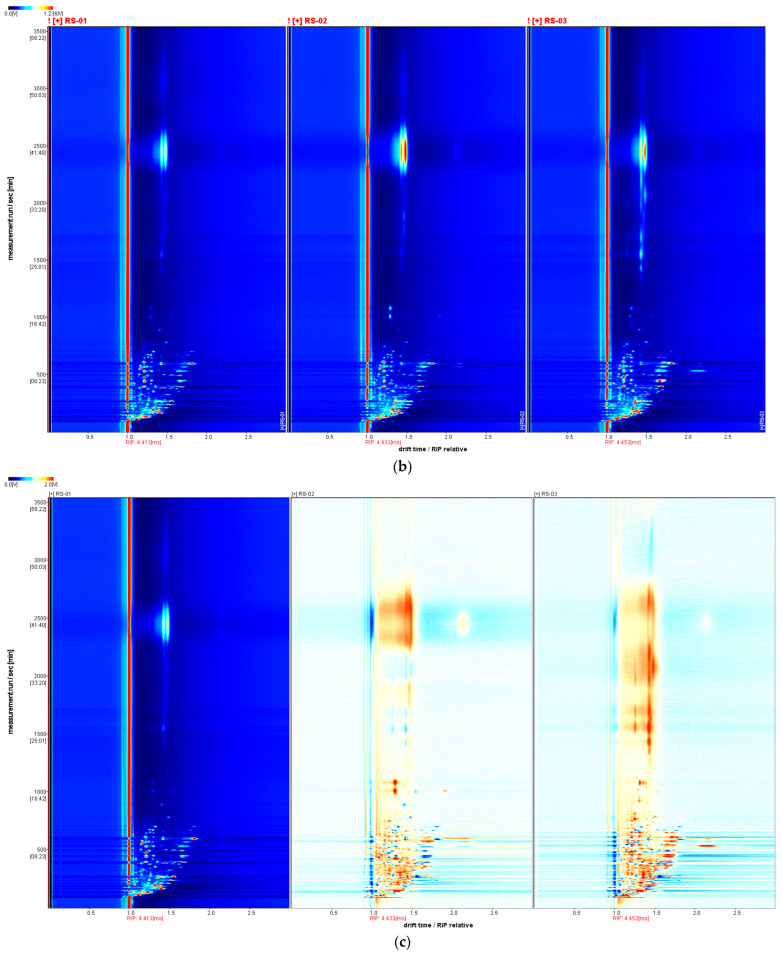
Three-dimensional (**a**), two-dimensional (**b**), and comparative map (**c**) of VOCs in different parts of ginseng powder.

The differences in VOCs from three different parts of ginseng (the main root, reed head, and fibrous root) powder were analyzed using GC–IMS. Based on the NIST and IMS databases, VOCs were determined by combining retention indexes (RI), retention times, and drift times. A total of 68 signal peaks was detected, and a total of 65 VOCs was identified. The results are presented in Table 1. Figure 1a shows the three-dimensional (3D) spectrum of GC–IMS, where the three axes stand for the mobility time (X-axis), retention time (Y-axis), and signal peak strength (Z-axis). In order to facilitate observation, a top view is shown for comparison. As depicted in Figure 1b, the graph features a blue background; the vertical axis denotes the retention time of the gas chromatogram, while the horizontal axis indicates the relative mobility time. The red vertical line at a horizontal coordinate of 1.0 corresponds to RIP (reactive ion peak, normalized). Each point on either side of RIP represents a volatile substance. The color represents the concentration of the substance; white means that the concentration is lower, and red means it is higher; the darker the color, the greater the concentration. It can be seen that there are certain differences in VOCs in the samples derived from different parts of ginseng powder.

To more intuitively compare the differences between samples, the spectrum of the RS-01 sample was chosen as the reference, and the spectra of other samples were subtracted to generate a comparative difference diagram for various samples, as depicted in Figure 1c. If the VOC content of the target sample and the reference is the same, the deducted background is white, whereas red means that the concentration of the substance in the target sample is higher than the reference, and blue means that the concentration of the substance in the target sample is lower than the reference. It can be seen that there are obvious differences in the contents of various VOCs in RS-01, RS-02, and RS-03, which is consistent with the results observed in the 3D spectrum and top view.

### 2.2. Differences in Characteristic VOCs Fingerprints

**Figure 2:**
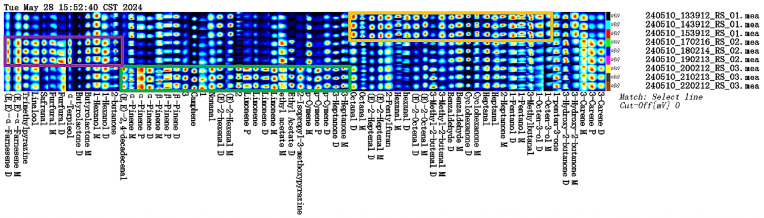
Fingerprints in different parts of ginseng powder.

In order to more intuitively identify differences between samples from different parts of ginseng powder, and the VOCs in the samples were further compared, and fingerprints were analyzed using the Gallery Plot plug-in, as shown in Figure 2. Each row represents all selected signal peaks in a sample, and each column represents the same VOCs in different samples. The color intensity represents the concentration of VOCs; the redder the color, the higher the relative content, and the bluer the color, the lower the relative content. Unidentified components are numbered 1–3. Through the comparative analysis of VOCs in samples RS-01, RS-02, and RS-03, the results show that Octanal, (E)-2-Heptenal, 2-Pentylfuran, Hexanal, (E)-2-Octenal, 3-Methyl-2-butenal, Benzaldehyde, Cyclohexanone, Heptanal, 2-Heptanone, 1-Pentanol, 3-Methylbutanal, 1-Octen-3-ol are high in main root (RS-01), as shown in the orange box. (E, E)-α-Farnesene, Trimethylpyrazine, Linalool, Safranal, Furfural, α-Terpieol, Butyrolactone, Hexanol, 2-butanone are high in reed head (RS-02), as shown in the purple frame. (E, E)-2,4-decadecenal, α-Pinene, β-Pinene, Camphene, Nonanal, (E)-2-hexenal, Limonene, Ethyl Acetate, 2-Isopropyl-3-methoxypyrazine, p-Cymene, 3-Heptanone are high in fibrous root (RS-03), as shown in the green box. It can be seen that the type and contents of VOCs can be used as an important indication to distinguish different ginseng parts powder.

### 2.3. Chemometric Analysis

#### 2.3.1. Principal Component Analysis

**Figure 3:**
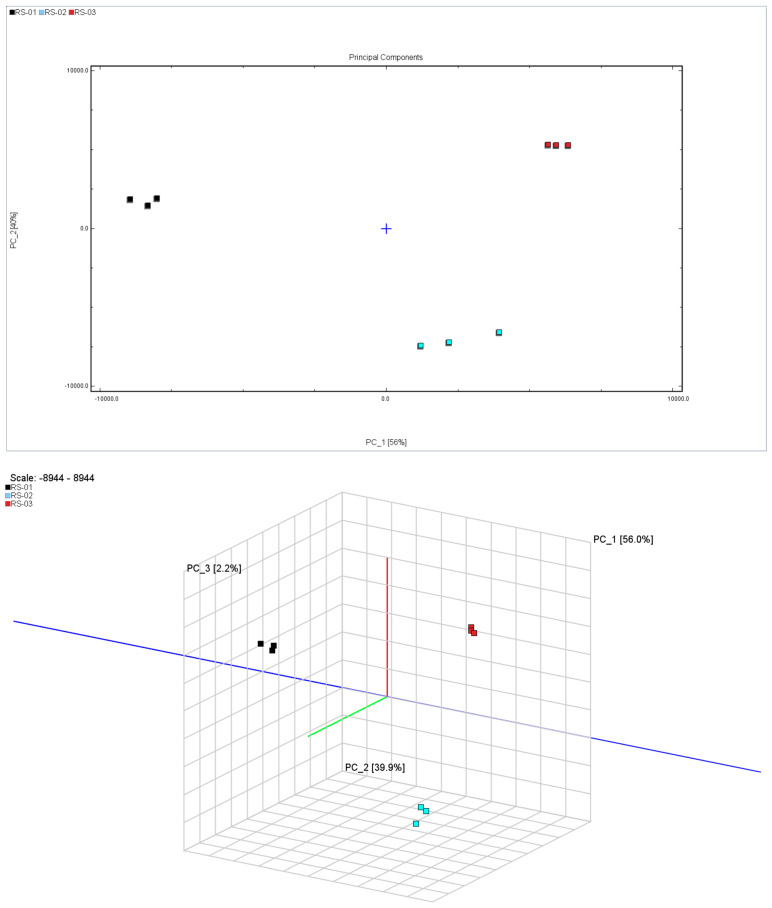
Results of the PCA analysis in different parts of ginseng powder.

Principal component analysis (PCA) is a data representation method used for feature extraction and dimensionality reduction. It reduces a large number of potentially relevant variables to a smaller number of uncorrelated variables, providing an overview of class separation, clustering, and outliers. Additionally, PCA facilitates model visualization, enhancing interpretability while mitigating subjective bias [22,23]. In order to distinguish the difference between the main root, reed head, and fibrous root, PCA was performed on the peak volume of 65 VOCs in 9 batches of samples, and the results are shown in Figure 3, where different colors represent different samples of ginseng powder. The distance between individual points represents the level of similarity, and the dispersion of the same points represents the homogeneity of the same sample. The contribution rate of PC1 is 56%, that of PC2 is 39.9%, and that of PC3 is 2.2%, and the cumulative contribution rate reaches 98.1%. The distance between the three samples is large, indicating that there were differences in VOCs in different parts of the ginseng powder.

#### 2.3.2. Euclidean Distance

**Figure 4:**
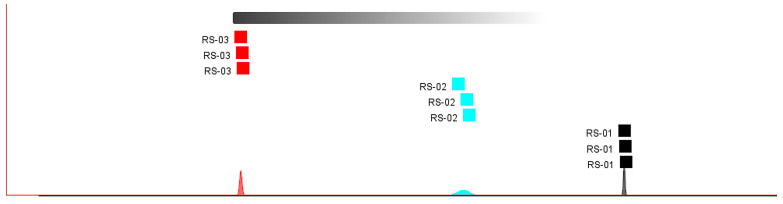
Fingerprint similarity based on Euclidean distance from different parts of ginseng powder.

The analysis of a fingerprint similarity map is a clustering analysis method based on Euclidean distance, which evaluates differences between samples, according to distance discrimination. This method takes into consideration the actual distance between two points in space or the natural length of the vector (from the point to the origin). The distance is directly proportional to the degree of similarity between the research objects [24]. Through the “nearest neighbor” fingerprint analysis of samples, the results showed the distances between ginseng powder samples of different parts can be clearly distinguished, and the results are shown in Figure 4. This indicated significant differences in VOCs between RS-01, RS-02, and RS-03 samples, which can be clearly distinguished. Among them, RS-03 and RS-01 are the furthest apart, which shows that the difference between them is the most significant. This is similar to the results of the PCA analysis.

#### 2.3.3. Partial Least-Squares Discriminant Analysis

**Figure 5:**
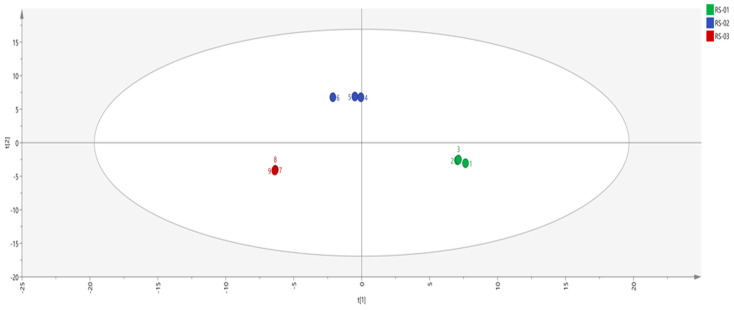
PLS−DA analysis of VOCs in different parts of ginseng powder.

Partial least-squares discriminant analysis (PLS-DA) is a supervised discriminant analysis method that can effectively distinguish observed values between groups, interpret observations, and predict corresponding variables [25,26]. The method’s reliability and predictive ability are evaluated using R^2^ and Q^2^. When R^2^ > 0.5 and Q^2^ > 0.5, this means that the method is reliable, and the closer R^2^ and Q^2^ are to 1, the stronger the predictive ability of the method is. In order to further distinguish the difference between main root, reed head, and fibrous root, the peak volume results of 65 VOCs from different samples were normalized, and PLS-DA scores were obtained with SIMCA software (version 14.1). The results are shown in Figure 5, where R^2^X = 0.957, R^2^Y = 0.995, and Q^2^ = 0.986. The distances between the three groups of samples were relatively large, indicating that there were obvious differences between the three groups, which was consistent with the conclusion drawn from PCA.

**Figure 6:**
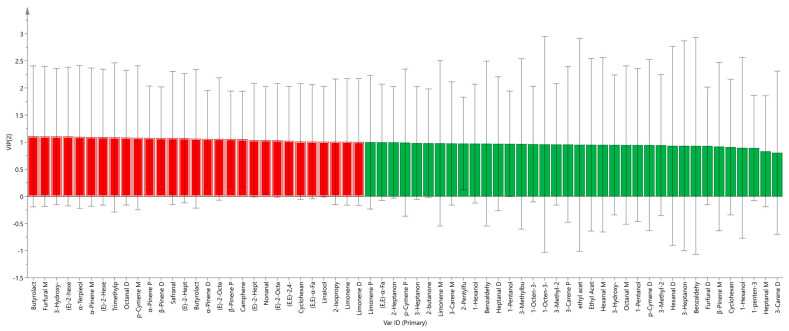
VIP values of the characteristic variables.

**Figure 7:**
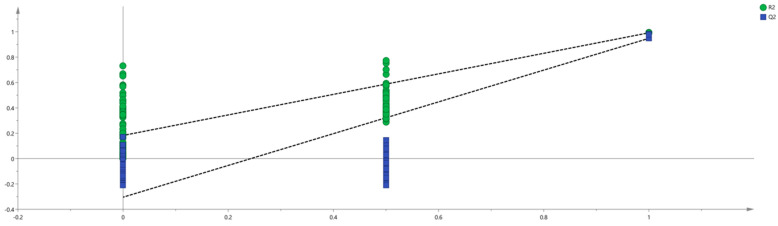
Permutation test results of VOCs in different parts of ginseng powder.

To measure the contribution of each variable, the variable important projection (VIP) of each volatile component variable was predicted based on the PLS-DA model. VIP is a parameter used to measure variable contribution to classification. Larger VIP values indicate higher contribution to classification. A VIP greater than 1 signifies substantial contribution to the discriminant model, serving as a marker screening criterion [27]. As depicted in Figure 6, a total of 29 VOCs exhibited significant contributions (indicated by the red bar chart), including (E, E)-α-Farnesene D, Safranal, α-Terpieol, Nonanal, 2-Isopropyl-3-methoxypyrazine, Linalool, (E)-2-Octenal M, (E)-2-Octenal D, p-Cymene M, Octanal D, Trimethylpyrazine, (E)-2-Heptenal D, (E)-2-Heptenal M, Cyclohexanone D, (E)-2-Hexenal M, (E)-2-hexenal D, 3-Hydroxy-2-butanone D, Furfural M, Butyrolactone M, β-Pinene D, β-Pinene P, Camphene, α-Pinene M, α-Pinene D, α-Pinene P, Butyrolactone D, Limonene D, Limonene, and (E, E)-2, 4-Decadecenal. These VOCs served as primary characteristic markers and were crucial in differentiating the samples of various parts of ginseng powder. Simultaneously, to avoid overfitting, we conducted 200 cross-validations to examine R^2^ and Q^2^ values. The steep slopes of the straight lines in Figure 7 indicate that the PLS-DA model was not overfitting (R^2^ = 0.219; Q^2^ = −0.261). The results demonstrated that the model had satisfactory predictive capability, meaning that the 29 VOCs could be used as reference indexes to identify the three parts of ginseng powder.

#### 2.3.4. Cluster Analysis (CA)

**Figure 8:**
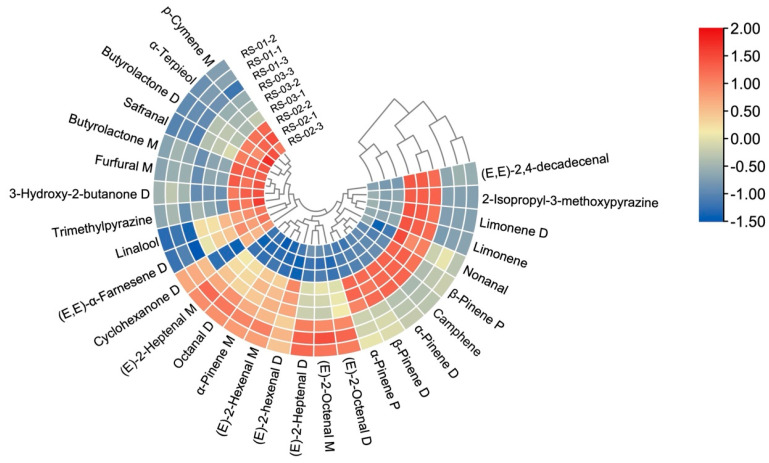
Cluster heat map of VOCs in different parts of ginseng powder.

Cluster analysis, a frequently employed statistical method, simplifies data aggregation and reflection by means of color changes. It can directly express data values through color depth and cluster samples with similar data [28]. To further clarify and screen out the characteristic VOCs that can be used to distinguish and identify the three different parts of ginseng powder, we imported 29 VOC peaks with VIP >1 from the three groups into TBtools software (version v2.119) for CA. The results are shown in Figure 8. The outer circle denotes the detected VOCs, while the column labels specify the ginseng sample names. The heat map displays relative values, with blue indicating lower values, and red indicating higher values. The results show that there are both similarities and differences in the content of characteristic VOCs from different parts of ginseng powder. Among them, the contents of (E)-2-Octenal M, (E)-2-Octenal D, Octanal D, (E)-2-Heptenal D, (E)-2-Heptenal M, (E)-2-Hexenal M, and α-Pinene M were higher in main root (RS-01) than in the other parts of ginseng powder. The contents of (E, E)-α-Farnesene D, Safranal, α-Terpieol, Linalool, p-Cymene M, Trimethylpyrazine, 3-Hydroxy-2-butanone D, Furfural M, Butyrolactone M, and Butyrolactone D were higher in reed head (RS-02) than in the other parts of ginseng powder. The contents of 2-Isopropyl-3-methoxypyrazine, β-Pinene D, β-Pinene P, α-Pinene D, α-Pinene P, Limonene D, Limonene, and (E, E)-2, 4-decadecenal were higher in fibrous root (RS-03) than in the other parts of ginseng powder. It is worth noting that the clustering of VOCs mostly occurs among their monomers, dimers, and polymers, indicating a strong correlation among these substances. The reason is that they may be linked together through various chemical reactions [29].

Figure 8 shows that the three ginseng parts can still be grouped and distinguished, which is consistent with the analysis results of GC–IMS fingerprint, PCA, and PLS-DA. In addition, the three ginseng parts can be broadly categorized into two groups, with the reed head (RS-02) in one, the main root (RS-01) and fibrous root (RS-03) in the other, suggesting similar characteristic VOC types and contents in the main root and fibrous root. Therefore, the 29 characteristic VOCs can effectively distinguish and identify different parts of ginseng powder and can be used as a landmark substance for identification.

## 3. Discussion

In the research of traditional Chinese medicine with the same homology of medicine and food, scholars have primarily focused on the active compounds in medicinal parts while overlooking non-medicinal parts. However, an increasing number of studies have demonstrated that non-medicinal parts of traditional Chinese medicine also contain substantial pharmacological elements, holding significant potential for development and utilization. For instance, the male flowers and leaves of new resource food *Eucommia ulmoides* exhibit a variety of pharmacological activities [30]. Therefore, through the rapid identification in different parts of plant Chinese medicine with the same homology of medicine and food, reasonable development, and utilization of its effective parts, substantial resource wastage can be mitigated, promoting sustainable resource use and development.

The major edible parts of ginseng, an important traditional Chinese medicinal herb with health care functions, are its roots and rhizomes (main roots, fibrous roots, and reed head). The price of ginseng varies by more than 10 times in three different parts. It is easy to distinguish the main root, reed head, and fibrous root from the appearance, but ginseng products are usually sold in powder form, and with different parts of the ginseng powder having a similar color and smell, it is difficult to distinguish with the naked eye or by smell. Therefore, using a simple and easy method to distinguish different parts of ginseng helps to improve their efficient utilization. As a straightforward and efficient technology with improved sensitivity and resolution, GC–IMS can precisely detect VOCs in ginseng products, showing promise in quality control, authentication, and personalized utilization. However, the application of GC–IMS in different parts of ginseng powder has not been previously reported either in China or internationally. Therefore, in this study, we carried out the content determination of VOCs in different parts of ginseng using this technique.

In our comparative analysis of VOCs in the main roots, fibrous roots, and reed head of ginseng, a total of 65 VOCs was identified using the GC–IMS method, which is rapid and sensitive, enabling the precise identification in different parts of ginseng powder. Through comparing the contents of VOCs in different parts of ginseng powder and performing PCA and Euclidean distance, we could clearly identify significant differences between them. This is mainly due to their different growth positions, developmental stages, and physiological functions in ginseng plants. The main root has a complex internal structure, including periderm, phloem, xylem, etc. It is the main part of ginseng plant, responsible for absorbing water and nutrients in the soil and supporting the growth of the whole plant. Fibrous roots have a relatively simple structure and have a large number of smaller roots growing on the main root. They are mainly responsible for increasing the absorption area of the root system and improving the absorption efficiency of water and nutrients of the plant. The reed head is the root part of ginseng, primarily connecting the main root and stem, storing nutrients, and performing other tasks.

We obtained 29 differential markers in different parts of ginseng powder using PLS-DA, and CA analysis, which has important guiding significance for rapid identification and quality control in different parts of ginseng powder. (E, E)-α-Farnesene, trimethylpyrazine, linalool, furfural M, safranal, α-terpineol, butyrolactone, and 3-hydroxy-2-butanone D were present in relatively high amounts in the reed head of ginseng. α-Farnesene is a sesquiterpene with an alkene carbon chain chemical structure, containing multiple unsaturated double bonds, which confers strong antioxidant properties [31]. The reed head of ginseng also exhibits adaptogenic effects, which include protecting myocardial cells, increasing coronary blood flow, enhancing immunity, improving the body’s energy metabolism, displaying antidiuretic properties, and inducing emesis [32]. The content of (E, E) -2, 4-decenoaldehyde, α-pinene D, β-pinene D, nonanal, limonene, 2-isopropyl-3-methoxypyrazine, and cymene was higher in the fibrous roots of ginseng. Fibrous roots have good antioxidant, immunomodulatory, anti-inflammatory, antidiabetes effects [33,34]. The content of octanal, (E)-2-heptenal, 2-pentylfuran, hexanal, (E)-2-octenal, 3-methyl-2-butenal, benzaldehyde, cyclohexanone, heptanal, 2-heptanone, amyl alcohol, and 1-octene-3-ol was higher in the main roots of ginseng. The main roots mainly have antioxidant, antiproliferative, and antigenotoxic activities [35]. In terms of market prices, the main roots of ginseng were the most expensive part, followed by the reed head of ginseng, whereas the fibrous roots of ginseng were the cheapest. Considering cost-effectiveness, anti-inflammatory, and anti-aging properties, the use of the reed head of ginseng is more advisable. Meanwhile, this study revealed certain differences in the contents of these components, which were similar in the main roots, fibrous roots, and reed head of ginseng. Among them, α-Pinene and β-Pinene, which have good antibacterial, antitumor, and antiviral activity [36], had the highest content in the fibrous roots of ginseng, and therefore consuming this part of the plant would derive antibacterial, antitumor, and antiviral benefits.

Present observations showed that GC–IMS can better characterize VOCs in the main root, reed head, and fibrous root of ginseng powder. The application of GC–IMS can better distinguish between three different parts of ginseng powder and provides a theoretical basis for the personalized development and utilization of them. This assay method can accurately identify the various parts of ginseng and provide a scientific basis and reference for a more efficient and safe utilization of ginseng resources in food and medicine.

## 4. Materials and Methods

### 4.1. Materials

Ginseng was collected from Songjiang Town, Fu song County, Baishan City, Jilin Province, China, kept in a box with ice, and transported to the laboratory within 24 h. The main root, reed head, and fibrous root were collected separately after cleaning.

The main roots, reed heads, and fibrous roots of ginseng were crushed by a grinder and sieved through an 80-mesh sieve; three kinds of sample powders were obtained, respectively, and the samples were numbered systematically as RS-01, RS-02, and RS-03.

### 4.2. Instruments and Equipment

Normal ketones included 2-butanone, 2-pentanone, 2-hexanone, 2-heptanone, 2-octanone, and 2-nononone (all analytically pure), Aladin Corporation, Shanghai, China. The equipment also included 20 mL headspace bottle, Shandong Haineng Scientific Instrument Co., Ltd., Shandong, China. MXT-5 capillary column (15 m × 0.53 mm, 1.0 μm), Restek, Mount EI, Pennsylvania, USA; FlavourSpec^®^ gas-phase ion mobility spectrometer, G.A.S, Dortmund, Germany; CTC-PAL 3 static headspace automatic sampling device, CTC Analytics AG, Basel, Switzerland; and VOCal data processing software (version 0.4.03), G.A.S, Dortmund, Germany.

### 4.3. Analysis with GC–IMS

Firstly, 0.5 g of ginseng samples (RS-01, RS-02, and RS-03) was accurately weighed, respectively, and placed in a 20 mL headspace bottle, incubated at 70 °C and 500 rpm for 20 min. The volume of the automatic headspace injection was 500 µL, and the temperature of the injection needle was 85 °C. Then the VOCs were subjected to a chromatographic column for separation at 60 °C for 30 min. Each sample was measured in three parallel groups. The column temperature and IMS was 60 °C and 45 °C, respectively. The ionization source was tritium source (3H), and the electric field strength was 500 V/cm. High-purity N_2_ (purity ≥ 99.999%) was used as the carrier and drift gas. The programmed pressure increase was as follows: an initial flow rate of 2.00 mL/min was maintained for 2 min, linearly increased to 10.00 mL/min within 8 min, linearly increased to 100.00 mL/min within 10 min, and maintained for 39 min. The total chromatographic running time was 59 min. The VOCs were identified based on the RIs of standard substances in the GC–IMS library.

### 4.4. Statistical Analysis

The GC retention index database (NIST 2020) and IMS mobility time database built into VOCal software were used for qualitative analysis of VOCs. The Reporter (Version 11.x) and Gallery Plot (Version 1.1.0.2) were used to plot the difference map (3D spectrum, 2D spectrum, and difference spectrum) and fingerprint of different samples. Principal component analysis (PCA) and cluster analysis (CA) was carried out using dynamic PCA (version 0.0.3) and TB tools (version v2.119). SIMCA (version 14.1) software was used for partial least squares discriminant analysis (PLS-DA) to calculate the variable important projection (VIP) value.

## 5. Conclusions

In this study, GC–IMS was used to analyze and identify VOCs in different parts of ginseng powder, resulting in the detection of 68 VOCs. Following qualitative analysis based on the database, the chemical characteristics of 65 VOCs were identified, including monomers and dimers of some substances. It is clear that the characteristic VOCs of different parts of ginseng powder are essentially the same, but their contents are different. We employed PCA and PLS-DA theories and methodologies for analysis, establishing a discriminant model capable of differentiating between the main roots, rhizomes, and fibrous roots of ginseng powder. The characteristic markers obtained by the VIP value and CA played a key role in ginseng powder identification, providing assistance for the selection of specific raw materials for ginseng products catering to different needs. Our method not only provides an important reference for the identification and the authenticity in different edible parts of ginseng but also ensures the improvement and enhancement of the quality standards of ginseng products in the market.

However, this study has limitations: there may be significant differences in VOC content in the main root, reed head, and fibrous root of ginseng due to the different growth environment (altitude, region, etc.) and growth cycle of ginseng. In addition, the GC–IMS database is not perfect, limiting the accurate quantitative analysis and comprehensive characterization of samples. Therefore, our next step is to collect ginseng from different regions with different growth years for comprehensive evaluation.

## Figures and Tables

**Table 1:** Results of the qualitative analysis of different parts of ginseng powder.

No	Compounds	CAS#	Formula	RI	Rt/s	Dt/ms	Odor Description
1	(E, E)-α-Farnesene D	C502614	C_15_H_24_	1487.1	2464.654	1.48579	Citrus, herbal, lavender, bergamot, myrrh, neroli, green
2	(E, E)-α-Farnesene M	C502614	C_15_H_24_	1485.7	2454.604	1.42258	Citrus, herbal, lavender, bergamot, myrrh, neroli, green
3	Safranal	C116267	C_10_H_14_O	1208.9	1077.330	1.28714	herbal, phenolic, tobacco, spicy
4	α-Terpieol	C10482561	C_10_H_18_O	1188.8	1014.845	1.28968	floral, lilac, terpenic
5	Nonanal	C124196	C_9_H_18_O	1104.8	790.476	1.49418	rose, citrus, strong oily
6	2-Isopropyl-3-methoxypyrazine	C25773404	C_8_H_12_N_2_O	1095.1	768.110	1.25062	mung, bean
7	Linalool	C78706	C_10_H_18_O	1086.2	748.099	1.21862	citrus, rose, woody, blueberry
8	(E)-2-Octenal M	C2548870	C_8_H_14_O	1065.5	703.366	1.32707	fresh cucumber, fatty, green herbal, banana, green leaf
9	(E)-2-Octenal D	C2548870	C_8_H_14_O	1065.5	703.366	1.82308	fresh cucumber, fatty, green herbal, banana, green leaf
10	p-Cymene M	C99876	C_10_H_14_	1020.4	615.079	1.21506	fresh, citrus, terpene, woody, spice
11	p-Cymene D	C99876	C_10_H_14_	1021.1	616.256	1.31284	fresh, citrus, terpene, woody, spice
12	p-Cymene P	C99876	C_10_H_14_	1017.8	610.371	1.72530	fresh, citrus, terpene, woody, spice
13	Octanal D	C124130	C_8_H_16_O	1013.9	603.308	1.81241	aldehyde, waxy, citrus, orange, fruity, fatty
14	Octanal M	C124130	C_8_H_16_O	1008.0	592.713	1.41418	aldehyde, waxy, citrus, orange, fruity, fatty
15	Trimethylpyrazine	C14667551	C_7_H_10_N_2_	1000.5	579.680	1.16306	roasted potato, peanut, cocoa, chocolate
16	1-Octen-3-ol D	C3391864	C_8_H_16_O	983.8	545.935	1.59477	mushroom, lavender, rose, hay
17	Benzaldehyde M	C100527	C_7_H_6_O	961.9	502.347	1.15250	bitter almond, cherry, nutty
18	Benzaldehyde D	C100527	C_7_H_6_O	961.9	502.347	1.46539	bitter almond, cherry, nutty
19	(E)-2-Heptenal D	C18829555	C_7_H_12_O	959.7	498.128	1.66210	spicy, green vegetables, fresh, fatty
20	(E)-2-Heptenal M	C18829555	C_7_H_12_O	957.8	494.613	1.25811	spicy, green vegetables, fresh, fatty
21	Heptanal D	C111717	C_7_H_14_O	899.9	396.891	1.69906	fresh, aldehyde, fatty, green herbs, wine, fruity
22	Heptanal M	C111717	C_7_H_14_O	902.7	401.109	1.35053	fresh, aldehyde, fatty, green herbs, wine, fruity
23	Cyclohexanone D	C108941	C_6_H_10_O	893.3	387.049	1.44426	strong pungent, earthy
24	Cyclohexanone M	C108941	C_6_H_10_O	892.3	385.643	1.15646	strong pungent, earthy
25	1-Hexanol M	C111273	C_6_H_14_O	869.7	354.709	1.32808	fresh, fruity, wine, sweet, green
26	1-Hexanol D	C111273	C_6_H_14_O	867.2	351.447	1.64167	fresh, fruity, wine, sweet, green
27	(E)-2-Hexenal M	C6728263	C_6_H_10_O	853.9	334.544	1.17881	green, banana, fat
28	(E)-2-hexenal D	C6728263	C_6_H_10_O	849.1	328.569	1.51598	green, banana, fat
29	Hexanal D	C66251	C_6_H_12_O	797.3	271.210	1.55279	fresh, green, fat, fruity
30	Hexanal M	C66251	C_6_H_12_O	800.8	274.795	1.26863	fresh, green, fat, fruity
31	1-Pentanol D	C71410	C_5_H_12_O	767.1	241.335	1.52334	balsamic
32	1-Pentanol M	C71410	C_5_H_12_O	768.4	242.530	1.24801	balsamic
33	3-Methylbutanal	C590863	C_5_H_10_O	668.3	164.979	1.40801	chocolate, fat
34	Ethyl Acetate D	C141786	C_4_H_8_O_2_	628.6	144.910	1.33439	fresh, fruity, sweet, grassy
35	ethyl acetate M	C141786	C_4_H_8_O_2_	633.5	147.226	1.09980	fresh, fruity, sweet, grassy
36	3-Hydroxy-2-butanone D	C513860	C_4_H_8_O_2_	715.4	196.240	1.32556	butter, cream
37	3-Hydroxy-2-butanone M	C513860	C_4_H_8_O_2_	722.2	201.644	1.07231	butter, cream
38	Furfural D	C98011	C_5_H_4_O_2_	831.1	307.393	1.33734	sweet, woody, almond, bready
39	Furfural M	C98011	C_5_H_4_O_2_	829.0	305.077	1.08507	sweet, woody, almond, bready
40	Butyrolactone M	C96480	C_4_H_6_O_2_	918.2	425.469	1.08166	cream, fat, caramel
41	β-Pinene M	C127913	C_10_H_16_	977.3	532.626	1.21289	resin, green
42	β-Pinene D	C127913	C_10_H_16_	976.0	529.856	1.29733	resin, green
43	β-Pinene P	C127913	C_10_H_16_	975.0	528.009	1.63287	resin, green
44	Camphene	C79925	C_10_H_16_	952.5	484.605	1.20951	woody, camphor
45	α-Pinene M	C80568	C_10_H_16_	932.7	449.650	1.21413	fresh, camphor, sweet, pine wood
46	α-Pinene D	C80568	C_10_H_16_	934.0	451.727	1.67103	fresh, camphor, sweet, pine wood
47	α-Pinene P	C80568	C_10_H_16_	934.3	452.246	1.72246	fresh, camphor, sweet, pine wood
48	3-Methyl-2-butenal D	C107868	C_5_H_8_O	786.0	260.170	1.35635	fruity
49	3-Methyl-2-butenal M	C107868	C_5_H_8_O	779.3	253.422	1.08907	fruity
50	1-penten-3-one	C1629589	C_5_H_8_O	667.2	164.389	1.30212	strong pungent odors
51	2-butanone	C78933	C_4_H_8_O	601.2	132.494	1.23571	fruity, camphor
52	2-Heptanone M	C110430	C_7_H_14_O	885.6	376.157	1.26226	pear, banana, fruity, slight medicinal fragrance
53	3-Heptanone D	C106354	C_7_H_14_O	889.3	381.315	1.62728	Fruity, Grass, Oil
54	Butyrolactone D	C96480	C_4_H_6_O_2_	917.2	423.868	1.29782	cream, fat, caramel
55	Limonene M	C138863	C_10_H_16_	1039.9	651.878	1.21338	lemon, sweet, orange, pine oil
56	Limonene D	C138863	C_10_H_16_	1038.1	648.350	1.29803	lemon, sweet, orange, pine oil
57	Limonene	C138863	C_10_H_16_	1038.5	649.055	1.6614	lemon, sweet, orange, pine oil
58	Limonene P	C138863	C_10_H_16_	1038.5	649.055	1.72667	lemon, sweet, orange, pine oil
59	2-Pentylfuran	C3777693	C_9_H_14_O	996.3	572.413	1.25131	bean, fruity, earthy, green, vegetable
60	3-Carene M	C13466789	C_10_H_16_	997.9	575.175	1.21279	citrus, lemon, woody
61	3-Carene D	C13466789	C_10_H_16_	995.3	570.204	1.30035	citrus, lemon, woody
62	3-Carene P	C13466789	C_10_H_16_	995.5	570.756	1.71712	citrus, lemon, woody
63	1-Octen-3-ol M	C3391864	C_8_H_16_O	985.8	550.111	1.15382	mushroom, lavender, rose, hay
64	3-Heptanone M	C106354	C_7_H_14_O	899.9	396.892	1.19904	fruity, grass, oil
65	(E, E)-2,4-decadecenal	C25152845	C_10_H_16_O	1331.6	1552.147	1.42880	cucumber, melon, citrus, pumpkin, nutty

Note: The suffixes M, D, or P denote the monomer, dimer, and polymer of the same substance. The software’s descriptions of the substances’ odors primarily draw from the databases of https://www.flavornet.org (accessed on 15 May 2024), https://www.femaflavor.org (accessed on 15 May 2024) and https://www.chemicalbook.com (accessed on 15 May 2024).

## Data Availability

Data are contained within the article.
